# Microbial keratitis: a community eye health approach

**Published:** 2015

**Authors:** Kieran S O'Brien, Thomas M Lietman, Jeremy D Keenan, John P Whitcher

**Affiliations:** Research Coordinator, Francis I Proctor Foundation and Department of Ophthalmology, University of California, San Francisco, USA.; Director and Professor, Francis I Proctor Foundation and Department of Ophthalmology, University of California, San Francisco, USA.; Associate Professor, Francis I Proctor Foundation and Department of Ophthalmology, University of California, San Francisco, USA.; Professor Emeritus, Francis I Proctor Foundation and Department of Ophthalmology, University of California, San Francisco, USA.

**Figure:**
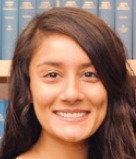
Kieran S O'Brien

**Figure:**
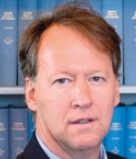
Thomas M Lietman

**Figure:**
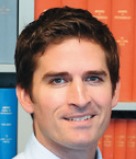
Jeremy D Keenan

**Figure:**
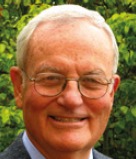
John P Whitcher

Microbial keratitis is an infection of the cornea. Corneal opacities, which are frequently due to microbial keratitis, remain among the top five causes of blindness worldwide. Microbial keratitis disproportionately affects low- and middle-income countries. Studies indicate that the incidence of microbial keratitis may be up to 10 times higher in countries like Nepal and India compared to the United States.

Rural agricultural communities in low-and middle-income countries face a particularly high burden from corneal blindness. The most common cause of microbial keratitis is infection following a corneal abrasion. People are at greater risk of corneal injuries from agricultural activities, manual labour, and domestic work, which can result in infections of the cornea through contact with contaminated objects. Microbial keratitis tends to affect people at younger ages, in their prime working years, compared to other causes of blindness (such as cataract), which generally affect older people

Rural communities in low- and middle-income countries face numerous obstacles in accessing appropriate treatment for microbial keratitis. Long delays in presentation and use of traditional medicines are common, increasing the risk of perforation and other complications that may result in vision loss. Patients with corneal ulcers may also face worse outcomes due to a lack of effective treatment options as well as an inability to afford medications when treatment is available. Opportunities for rehabilitation through surgical procedures are also limited by a lack of donor corneas for transplants.

Even when appropriate medical care is available, the corneal scarring that accompanies healing often results in visual impairment, despite successful antimicrobial treatment. Trials comparing antimicrobials for microbial keratitis generally have been unable to discern differences in visual acuity after treatment. An exception is that natamycin has been shown to be more effective than voriconazole for fungal corneal ulcers. Studies trialling adjunctive therapies with agents, such as topical corticosteroids, to reduce scarring, also have been largely unable to demonstrate major differences in visual outcomes in bacterial keratitis.

**Figure:**
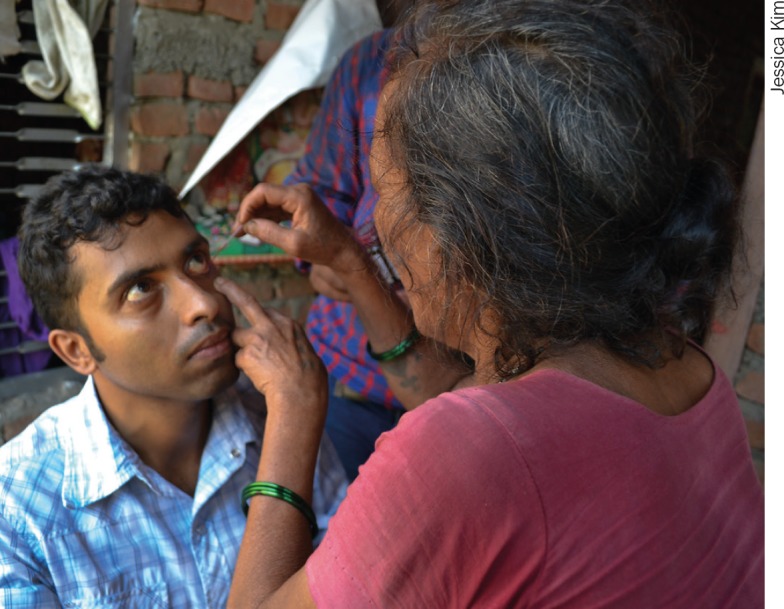
A community health volunteer practises applying fluorescein to detect corneal abrasions. NEPAL

Given the limitations associated with available treatment options, secondary prevention (i.e. the prevention of visual impairment in someone with a corneal injury and/or infection) may be the best option for reducing vision loss associated with microbial keratitis.

A series of studies in Southeast Asia suggested that antimicrobial ointment applied soon after a corneal abrasion could dramatically reduce the incidence of microbial keratitis. The Bhaktapur Eye Study in Nepal was the first of these to show promising results for microbial keratitis prevention programmes at village level. In this study, primary eye care workers from the community were trained to diagnose corneal abrasions with fluorescein strips and a blue torch. They then provided topical chloramphenicol to all patients with a corneal epithelial defect. This study found that only 4% of patients treated for a corneal abrasion developed a corneal ulcer, and that an ulcer only developed if the antibiotic was applied more than 18 hours after the eye trauma.

**ABOUT THIS ISSUE**

This issue of the *Community Eye Health Journal* focuses on micobial keratitis – corneal ulceration caused by microorganisms – which isa major cause of unilateral (and some cases of bilateral) corneal blindness, particularly in rural low-resource settings. The aim of the issue is to promote good practice in preventing, diagnosing and treating microbial keratitis. There are also practical articles on how to take a corneal scrape in microbial keratitis and the indications and procedure for tarshorrhaphy. We hope you find the articles of help in your work and we look forward to receiving any comments you may have.

A similar study conducted in Bhutan corroborated the Nepal study's findings, and suggested that a microbial keratitis prevention programme may be effective even in isolated rural areas. In Myanmar, low rates – much lower than previous estimates – of bacterial and fungal ulcers were observed after the institution of the village eye worker programme. In a trial conducted in South India in individuals with corneal abrasions, those randomised to antibiotic prophylaxis had low rates of corneal ulcers, similar to rates observed in patients randomised to antibiotic plus antifungal prophylaxis, suggesting that antibacterial prophylaxis alone might prevent both bacterial and fungal infections.

These studies demonstrated that village health workers can be trained to diagnose corneal abrasions and provide prophylactic treatment, and suggested that this simple intervention might be effective.

These studies also indicate that the following simple tools may be used to identify and prevent microbial keratitis.

1. **Fluorescein dye.** Applied to the eye using sterile strips or solution, fluorescein will stain corneal epithelial defects/abrasions.
2. **Blue torch.** A blue light shone onto the cornea with fluorescein dye will highlight a corneal abrasion, which is visible as a bright green area.
3. **Loupes.** Magnifying loupes are helpful in determining the existence of a corneal abrasion.
4. **Prophylaxis.** Once a corneal abrasion is identified, antibiotic and antifungal ointments should be applied three times a day for 3 days to prevent infection.
5. **Education.** Health education campaigns inform local community members about corneal infections and encourage them to seek care in the event of ocular injury.

As infectious ocular diseases decline, microbial keratitis continues to be a major cause of vision loss globally. While the continued exploration of treatment options for corneal ulcers is essential, we must also focus efforts on opportunities for prevention. In low- and middle-income countries, the prevention of microbial keratitis is a promising intervention for reducing corneal blindness. A large community randomised trial (Village Integrated Eye Worker trial, NIH-NEI U10EY022880) examining corneal ulcer prevention by trained village-level health workers is currently underway in Nepal. Similarly, another study in south India will further examine corneal ulcer education programmes.

Looking forward, with increased awareness and implementation of preventive strategies, it should be possible to reduce the burden of corneal blindness worldwide.
